# Morphogenetic identification and enzymatic determination of local isolate fungi associated with mosquitoes at Assiut Governorate

**DOI:** 10.1186/s12866-026-04974-0

**Published:** 2026-05-23

**Authors:** Sina M. Morsy, Farouk A. Abdel-Galil, Ahmed I. A. Farghal, Mohamed A. Abdel-Nasser, Sara E. Mousa

**Affiliations:** Plant Protection Department, Faculty of Agriculture, Assiut University, Assiut, Egypt

**Keywords:** Local Fungal Isolates, Morphological and Molecular Identification, And Enzymatic Detection

## Abstract

**Background:**

Mosquitoes pose a significant threat as vectors for numerous human and animal diseases, particularly in tropical and subtropical regions, including Egypt. Given the prevalence of mosquito-borne diseases and the documented high densities of *Culex pipiens* in Assiut Governorate, there is a critical need for environmentally friendly mosquito management strategies. This study aimed to identify the morphogenetic and determine the enzymatic potential of native fungal isolates associated with mosquitoes in Assiut Governorate.

**Results:**

Eight distinct fungal species were successfully characterized based on their morphological traits and ITS rDNA sequence data. These species were identified as *Alternaria tenuissima*, *Trichoderma hamatum*, *Purpureocillium lilacinum*, *Geotrichum candidum*, and four *Fusarium* species (*F. oxysporum*, *F. solani*, *F. equiseti*, and *F. incarnatum*). The results of enzymatic screening emphasize that twenty-two fungal isolates belonging to eight species had the biochemical machinery substantial for cuticle degeneration, with various levels of phospholipase, lipase, protease, and chitinase activities. The strongest synergistic enzymatic profiles were notably displayed by *P. lilacinum* and *F. equiseti*.

**Conclusion:**

The extensive morphological and molecular description and enzymatic detection provide crucial promotion for evaluating the potential of these local fungal isolates for future sustainable insect pest management in the specific ecological context of Assiut Governorate.

## Background

Mosquitoes are vectors of several deadly and dangerous human and animal diseases like Filariasis, lymphatic filariasis, Malaria, Sindbis virus, Chikungunya, Dengue, Japanese encephalitis, Usutu virus, Tahyna virus, Batai virus, Japanese encephalitis virus, West Nile virus, and Zika virus [17]. Billions of human lives are threatened by mosquito-borne diseases, especially in tropical and sub-tropical zones [1, 30].

In Egypt, several mosquito genera are present; most species in these genera serve as significant disease vectors. Mosquitoes include *Culex*, *Anopheles*, *Aedes*, *Culiseta*, and *Uranotaenia* [6]. Also, Sowilem et al. [27] recorded several mosquito species with various densities in Assiut. The mosquito species *Culex pipiens* is more abundant. It should be kept in mind that Assiut Governorate villages had been enrolled as endemic foci in the international and national programs of elimination of lymphatic filariasis [5]. An abundance of vectors could be explained by environmental and biological features supporting vector breeding, as in El Nikhila, El-Matiaa villages, and the Sahel Seleem district of Assiut Governorate. Such features include the presence of agricultural drain water because of changes in the irrigation system in Upper Egypt, which was formed due to a poor drainage system [3]. In addition, dense vegetation covering throughout the periphery of water banks not only provides favorable ovipositional sites but also protects mosquito larvae from predacious insects and fish [5]. Therefore, it must be pointed out to what extent the danger of the presence of these pools. It is essential to discover and keep investigating environmentally friendly methods to develop an alternate strategy for larval management to fight the expansion of mosquitoes’ habitat predicted in certain geographical areas in association with the occurring climatic changes.

There is a wide range of entomopathogenic fungi (EPF), and many, especially local fungi strains, have been proposed as microbial biological control agents (MBCAs). Native fungal isolates offer a better alternative for biological control of vectors as they may be better adapted to both kill local insects and survive local environmental conditions [24]. The first key step to start developing this tool is therefore to isolate and assess the capacity of local fungi. Isolating and assessing local fungi is important for developing programs to control insects. Entomopathogenic fungi produce destructive enzymes such as proteases, chitinase, and lipases, which degrade insect cuticles and aid in infection. These enzymes are vital for fungal attachment, germination, and colonization. Understanding the link between enzyme production and fungal virulence can help identify potent isolates for new bio-products [23].

So, the present study focused on identifying and describing indigenous fungal isolates associated with mosquitoes in agricultural drainage water at Assiut Governorate. This study involved morphological and molecular characterization of these fungal species, using both macroscopic and microscopic features, and confirming their identity through DNA sequencing.

Additionally, to determine the possible secretion of enzymes, particularly proteases, lipases, chitinases, and phospholipases, was detected.

## Materials and methods

### Source of entomopathogenic fungi (EPF)

In the present study, 22 fungal isolates belonging to 8 species were selected as potential entomopathogenic fungal isolates. These isolates were previously collected from agricultural drainage water and are associated with the immature stage of mosquito species.

Agricultural drainage water containing mosquito larvae and associated insects was collected from Assiut Governorate and filtered. 50 mg/L chloramphenicol was added to the samples, and sterile sesame seed baits were applied. The samples were then incubated at 28±2°C, 75±5%RH under dark conditions for 3–7 days. The resulting fungi were transferred to two culture media and purified using PDA and SDA agar. Pure isolates were obtained from the baits and insect stages [2, 14]. It was deposited in the Assiut University Moubasher Mycological Center (AUMMC) (Table 1).

**Table 1:** Taxon scientific name and AUMC accession number of locally isolated eight fungal species

No	Taxon scientific name	AUMC accession number
1	*Alternaria tenuissima*	AUMC 16518
2	*Trichoderma hamatum*	AUMC 16511
3	*Fusarium oxysporum*	AUMC 16512
4	*Fusarium solani*	AUMC 16514
5	*Fusarium equiseti*	AUMC 16515
6	*Fusarium incarnatum*	AUMC 16516
7	*Purpureocillium lilacinum*	AUMC 16504
8	*Geotrichum candidum*	AUMC 16517

### Morphological identification

Pure fungal cultures (5–7 days old) were inoculated onto four different media: Sabouraud Dextrose Agar (SDA) [19], Oatmeal Agar (OA) [26], and Potato Sucrose Agar (PSA). Macroscopic features, including colony color, growth rate, morphology, and conidial pigmentation, were recorded at 4, 5, and 7 days post-inoculation. For microscopic analysis, slide cultures were prepared from 5- to 7-day-old colonies and stained with aniline blue. Morphological features such as hyphal arrangement, size, and conidial shape were examined. Species identification was performed using established taxonomic keys, including those by De Hoog et al. [4], Moubasher [15], and Luangsa-Ard et al. [12].

### Molecular identification

#### DNA extraction

Tiny fragments of fungal mycelia from the 7-day-old colony of different isolates, grown on the cultivation media PDA, were individually collected and moved to the 2mL-Eppendorf. DNA was extracted from fungal cultures using the acetyltrimethylammonium bromide procedure combined with an extra polyethylene glycol precipitation [21].

A slightly modified version of the Cetyltrimethylammonium bromide (CTAB) method was used to extract genomic DNA from fungal isolates. In a nutshell, roughly 100 mg of fresh fungal mycelia were ground into a powder. The powder was put into a microcentrifuge tube with 700 µL of CTAB extraction buffer (2% CTAB, 100 mM Tris-HCl, pH 8.0, 20 mM EDTA, and 1.4 M NaCl) that had been preheated to 65°C. After 30 to 60 minutes of incubation at 65 °C, an equivalent volume of chloroform: isoamyl alcohol (24:1) was added, and the mixture was centrifuged for 10 minutes at 12,000 rpm. Cold isopropanol was added to precipitate the DNA after the upper aqueous phase was recovered. The resultant pellet was reconstituted in 50 µL of sterile DNase-free water after being air-dried and cleaned with 70% ethanol. The purity and concentration of the DNA were verified using a spectrophotometer.

#### PCR conditions

The PCR reaction was conducted by the standardized primers ITS1 (5'TCCGTAGGTGGT GAACCTTGCGG3') and ITS4 (5'TCCTCCGCTTATTGATATGC3') for DNA amplification in a 50μL reaction [8, 13]. The DNA base 1μL, each primer1μL, PCR master mix 25μL, and distilled water 22μL were applied to the PCR tube. Then the PCR amplification was carried out using the following sequence: one round of amplification consisting of denaturation at 95 °C for 15 min followed by 30 cycles of denaturation at 95 °C for 20secs, annealing at 50 °C for 40secs, and extension at 72 °C for 1 min, with a final extension step of 72 °C for 5min.

#### Sequencing, alignments, and phylogenetic analysis

DNA sequencing for this study was exclusively performed by Macrogen, located in Seoul, South Korea. The resulting sequences were subsequently aligned with known ITS1 and ITS4 sequences of each fungal isolate species in the rDNA in the GenBank database using the basic local alignment search tool (BLAST) at the National Center for Biotechnology Information (NCBI) (https://blast.ncbi.nlm.nih.gov/Blast.cgi?PROGRAM=blastn&PAGE_TYPE=BlastSearch&LINK_LOC=blasthome), and the percent homology scores were generated to identify our fungal isolates. Then, those isolations were submitted to the National Centre for Biotechnology Information (GenBank/NCBI) to acquire their unique accession numbers (http://www.ncbi.nlm.nih.gov). For subsequent analysis, the DNA sequences underwent initial alignment using CLUSTALW's default settings [29]. Phylogenetic reconstructions were carried out using MEGA software version 12.0 [10], employing both Minimum Evolution (ME) and Neighbor-joining (NJ) methods. To assess the robustness of these reconstructions, 1000 bootstrap iterations were applied [7]. Finally, sequence divergences were determined using Kimura 2-parameter distances [9].

### Detection of extracellular enzyme activity

The enzymatic potential of twenty-two fungal isolates belonging to eight species was evaluated. Protease, chitinase, lipase, and phospholipase activities were screened using modified Sucrose-Free Czapek’s Agar (Cz) [28]. The basal medium consisted of (g/L): NaNO_3_ 2.0, KCl 0.5, K_2_HPO_4_ 1.0, MgSO_4_·7H_2_O 0.5, ZnSO_4_ 0.01, CuSO_4_ 0.01, FeSO_4_·7H_2_O 0.01, and agar 20.0. Each enzyme’s specific substrate was added individually to the basal medium to induce activity. For each test, non-inoculated plates were used as negative controls to ensure that the degradation zones were solely due to fungal activity. Also, *Beauveria bassiana* AUMC17426 was used as a positive control. The enzymatic activity was assessed semi-quantitatively by measuring the diameter (mm) of the clear or precipitation zone around the fungal colony after incubation.

#### Lipase

Lipolytic activity was assessed using Czapek’s agar supplemented with 10 mL of separately sterilized Tween 80. The formation of a visible precipitate served as an indicator of lipolysis. The depth of the precipitate (mm) was recorded following a 15-day incubation period at 28±2°C, 75±5%RH under dark conditions.

#### Protease

Proteolytic activity was screened in the basal medium supplemented with 15% sterile skimmed milk. After 17 days of incubation at 28±2°C, 75±5%RH under dark conditions, the clear zone resulting from casein hydrolysis was measured (mm) as an indicator of protease secretion.

#### Chitinase

Screening of chitinase for fungi isolates using the basal medium containing 0.5% colloidal chitin. After inoculation, the plate was incubated for 72 h at 28±2°C, 75±5%RH under dark conditions, flooded with 0.5mM iodine solution for 20 min, and rinsed with water.

Enzyme secretion of fungi was detected based on the formation of a clear zone that appeared around the colonies.

#### Phospholipases

To detect fungal isolates' phospholipase enzyme activity, the medium described by Muhsin et al. [16] was employed. This medium contained (per liter): 5.0g yeast extract, 5.0g Tryptone, 20.0g proteose peptone, 5.0g sodium chloride, and 15 g agar. To the molten medium, 80mL of a 1:1 egg yolk to 0.85% saline solution was added.

Ten mL aliquots of the prepared medium were aseptically dispensed into 15-mL test tubes. Each tube was then inoculated with 50µL of a fungal spore suspension derived from a 7-day-old culture. The inoculated tubes were incubated at 25 °C for 14days to allow for the development and detection of phospholipase activity.

### Statistical analysis

Data were expressed as the Mean ± Standard Deviation (SD). Due to the unbalanced nature of the experimental design (unequal number of replicates per species), a one-way analysis of variance (ANOVA) was performed to evaluate the differences in enzymatic activities. To account for the unequal sample sizes, Duncan’s Multiple Range Test with Kramer’s adjustment was employed for multiple comparisons of the means at a significance level of P < 0.05. Also, Significant differences between means are denoted by different lowercase letters in the figures. All statistical computations were generated using SAS Version 9.1 [22] and high-resolution graphical representations by using GraphPad Prism Version 10.

## Results

### Morphological identification and description of fungi

#### *Alternaria tenuissima* AUMC 16518

**Fig. 1:**
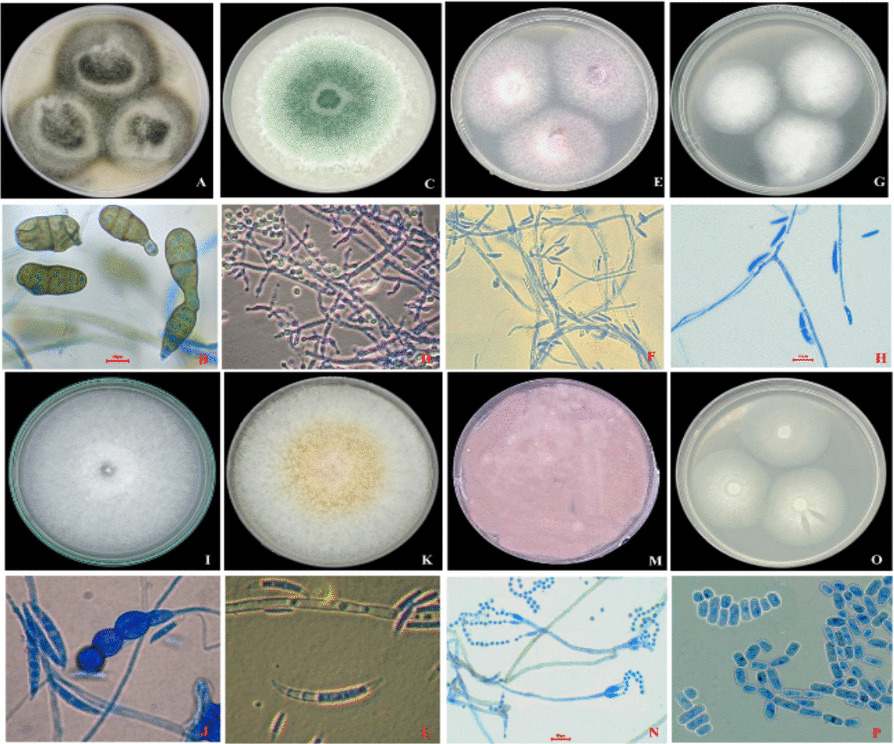
Description of prevalent fungal species: **A**, **B** *Alternaria tenuissima*; **C**, **D** *Trichoderma hamatum*; **E**, **F** *Fusarium oxysporum;* **G**, **H*** Fusarium solani;* **I**, **J*** Fusarium equiseti;* **K**, **L*** Fusarium incarnatum;* **M**, **N** *Purpureocillium lilacinum*, and **O**, **P*** Geotrichum candidum*

*Alternaria tenuissima* colonies grow quickly on malt extract agar at 25 °C, filling the plate within 7 days. Its conidiophores are erect, pale to mid-brown, and up to 100µm long, bearing 1-6conidial scars. Conidia, measuring up to 100x5-12µm, are solitary or in short chains, obclavate or elliptical, pale to mid-brown, smooth to finely verruculose, and have 2–10 transverse and several longitudinal/oblique septa. A distinctive feature is their beak, which can be up to half the conidium's length and often swollen at the apex due to conidial scars; some may lack a beak, thus excluding it from *A. brassicae,* which is characterized by much larger spores (often exceeding 150 μm). (Fig. 1A, B).

#### *Trichoderma hamatum* AUMC 16511

Colonies growing rapidly filling the whole plate (9 cm) after 3 days at 25 °C, on oatmeal agar (OA), at first thin, translucent, later appearing floccose patches especially towards the margin of the colonies, at first white then acquiring green colocendiophials long stout and thick, highly branched with side branches mostly short commonly conidiophonyphal elongations, with straight a flexuous, whip-like or occasionally coiled apices; phialides crowded short and stout; conidia cylindrical with rounded ends, smooth-walled, 3.3X2.5um (Fig. 1C, D).

#### *Fusarium oxysporum* AUMC 16512

Colonies fast-growing, reaching 6.0cm in diameter after 5days on potato sucrose agar (PSA) at 25 °C, white, sometimes peach with a purple or violet tinge; mycelium sparse to abundant and floccose; microconidia abundant, single-celled, elliptical, straight to curved, 4–11×2-3um, formed from simple short phialides, arising laterally on the hyphae or from short sparsely branched conidiophores, never forming chains; macroconidia abundant, or sparse in some isolates, formed from simple phialides borne on branched conidiophores, usually 3–5-septate, slightly sickle-shaped, thin-walled, with a pointed apical cell and a foot-shaped basal cell, 27–60×3-5um, chlamydospores abundant, formed singly but occasionally in pairs or chains, terminal and intercalary (Colour plate). *F. oxysporum* may be mistaken with *F. moniliforme* var. *subglutinans*, but the latter has microconidia borne on polyphialides, and chlamydospores are not produced. (Fig. 1E, F).

#### *Fusarium solani* AUMC 16514

This *Fusarium* species exhibits rather fast-growing colonies on PSA or OA, appearing green to bluish-brown with abundant cream to buff conidial slime. Microconidia are typically abundant (8–16×2–4-5µm), produced on elongate conidiophores. Macroconidia are variably produced on branched conidiophores, usually moderately curved, mostly indistinctly 3-septate (28–42×4–6µm), with blunt apical and indistinctly pedicellate basal cells. Chlamydospores (6–10µmdiam.) are commonly produced singly or in pairs, terminally, laterally, or intercalary, often originating from both macro- and microconidia (Fig. 1G, H).

#### *Fusarium equiseti* AUMC 16515

This fungal species forms fast-growing colonies, reaching 7.2cm in diameter in 5days at 25 °C on potato sucrose agar (PSA), appearing pale pink to peach with bright orange sporodochia. It produces abundant, 1–5-septate macroconidia (9-34x2.1–3.7.7µm) from penicillately branched conidiophores, characterized by an elongated pointed apical cell and a foot-shaped basal cell, but lacks microconidia. It is heterothallic, forming globose, black perithecia with verrucose walls, containing clavate asci with 8elliptical to fusoid ascospores that have 1-3septa; chlamydospores are sparse or absent (Fig. 1I, J).

#### *Fusarium incarnatum* AUMC 16516

This *Fusarium* species grows rapidly, reaching ~6cm diameter in four days on PSA or OA at 25 °C, forming floccose, whitish to buff-brown aerial mycelium and peach-colored undersides. The fungus produces fusiform conidia that are typically 3–5-septate and measure 17–40(−50)×3.0–4.5.0.5µm, formed from slender, sympodial proliferating phialides. Microconidia are absent, sparse, globose, and intercalary chlamydospores (5–10µmdiam.) (Fig. 1K, L).

#### *Purpureocillium lilacinum* AUMC 16504

Colonies of *P. lilacinum* on MEA grow moderately fast, initially appearing white before shifting to a characteristic lilac or vinaceous-pink hue upon sporulation. Microscopically, the conidiophores are verticillate, bearing clusters of ovate to cylindrical phialides (6–9×2.5–3 µm) with distinct short necks. The ellipsoidal, smooth-walled conidia (2–3×2–4 µm) are produced in long, dry chains. This species is distinguished from *Paecilomyces* by its unique phylogenetic placement and thermotolerance [12]. (Fig. 1M, N).

#### *Geotrichum candidum* AUMC 16517

Colonies on Sabouraud-glucose agar (SDA) are fast-growing, white, and velvety with a notable fruity odor. The mycelium consists of dichotomously branched hyphae (7–11 µm wide) that fragment into cylindrical, smooth-walled arthroconidia (6–12×3–6 µm). The absence of blast conidia and the presence of ascending arthroconidial chains differentiate this isolate from related genera such as *Trichosporon* [4] (Fig. 1O, P).

### Molecular identification of fungi isolates

This study conducted a molecular and phylogenetic analysis of eight different fungal species. To confirm the classification of each fungus, the internal transcribed spacer (*ITS*) region of its rDNA was sequenced. These sequences were then used for phylogenetic reconstruction and compared against reference data in the GenBank repository.

The analysis validated the original identification of each fungus. Phylogenetic analyses demonstrated that all isolates exhibited high sequence homology with, and formed monophyletic clades containing, conspecific strains from the database. This confirmation was further supported by pairwise genetic distance calculations, which provided a quantitative assessment of evolutionary divergence and identified the most closely and distantly related taxa.

The same methodology was applied to all eight fungal species: the ITS1/ITS4 region was sequenced, the sequence was submitted to GenBank, and its evolutionary relationship to other fungi was analyzed using Minimum Evolution and Neighbour-Joining methods.

#### *Alternaria tenuissima* AUMC 16518

##### Sequence

A 534bp fragment (GenBank accession PV050363.1) was obtained, characterized by a higher Adenine-Thymine (A+T) content (53.56%) than Guanine-Cytosine (G+C) content.

##### Phylogenetic analysis

**Fig. 2:**
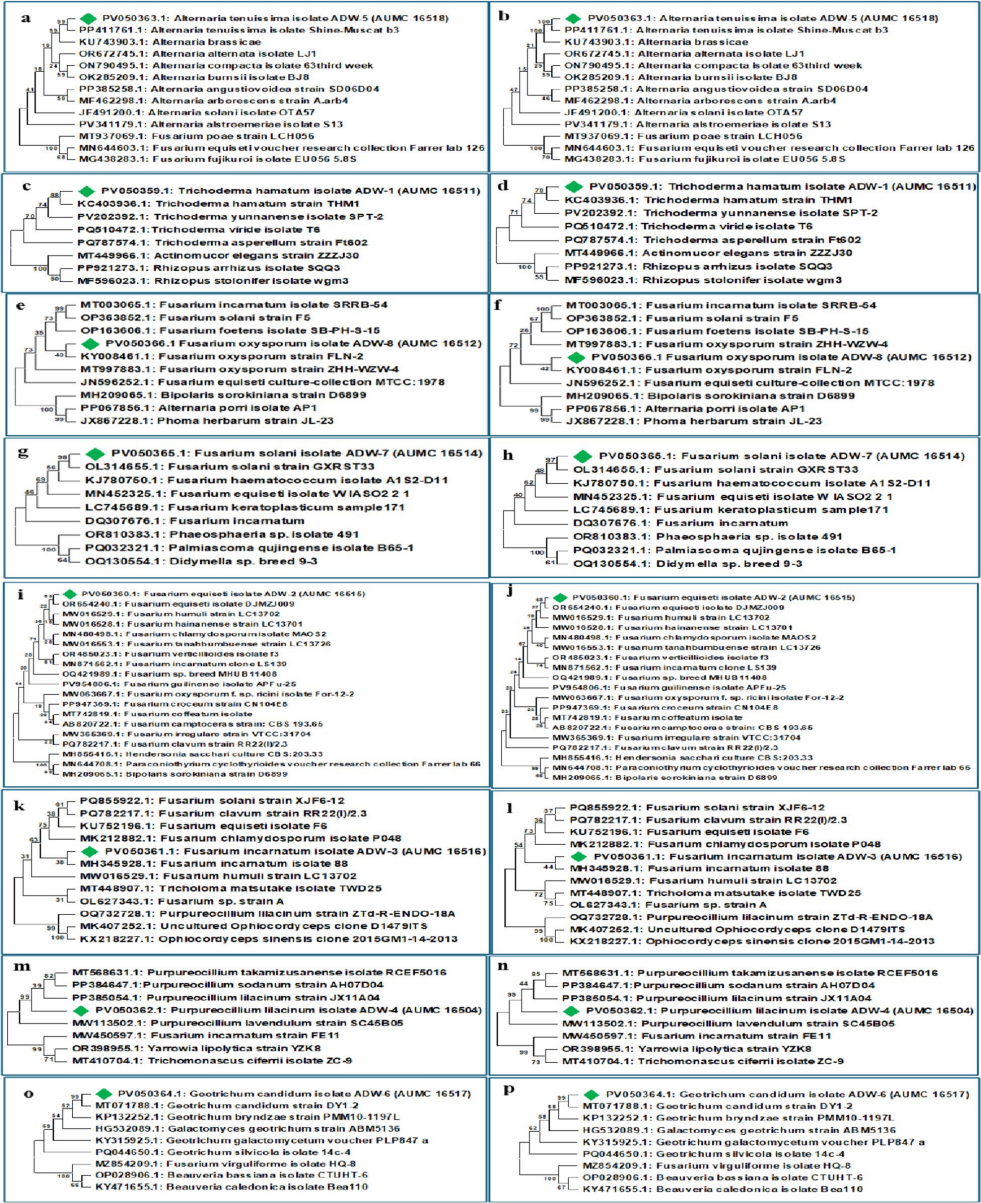
Phylogenetic trees illustrating the taxonomic placement of the fungal species endophytes based on (*ITS* gene) sequences. The left column (**a**, **c**, **e**, **g**, **i**, **k**, **m**, **o**) shows trees constructed using the Minimum Evolution (ME) method, and the right column (**b**, **d**, **f**, **h**, **j**, **l**, **n**, **p**) shows trees constructed using the Neighbor-Joining (NJ) method. The panels represent the analysis of: (**a**, **b**) *Alternaria tenuissima* AUMC 16518; (**c**, **d**) *Trichoderma hamatum* AUMC 16511; (**e**, **f**) *Fusarium oxysporum* AUMC 16512; (**g**, **h**) *Fusarium solani* AUMC 16514; (**i**, **j**) *Fusarium equiseti* AUMC 16515; (**k**, **l**) *Fusarium incarnatum* AUMC 16516; (m, n) *Purpureocillium lilacinum* AUMC 16504; (o, p) *Geotrichum candidum* AUMC 16517

It showed a direct relationship with *A. tenuissima* (PP411761.1) strains from 9species of the *Alternaria* family and three species of the Hypocreales order acting as an out-group (Table 2 and Fig. 2a, b).

**Table 2:** Taxa and GenBank accession numbers for the phylogenetic analysis of *Alternaria tenuissima* based on ITS sequences

No	Species	Accession number
1	*Alternaria tenuissima*	PV050363.1
2	*Alternaria tenuissima*	PP411761.1
3	*Alternaria brassicae*	KU743903.1
4	*Alternaria alternata*	OR672745.1
5	*Alternaria alstroemeriae*	PV341179.1
6	*Alternaria angustiovoidea*	PP385258.1
7	*Alternaria compacta*	ON790495.1
8	*Alternaria burnsii*	OK285209.1
9	*Alternaria arborescens*	MF462298.1
10	*Alternaria solani*	JF491200.1
11	*Fusarium poae*	MT937069.1
12	*Fusarium equiseti*	MN644603.1
13	*Fusarium fujikuroi*	MG438283.1

##### Genetic distance

It was the closest relatives' distance of 0.0000 to both *A. tenuissima* (PP411761.1) and *A. brassicae* (KU743903.1). While the most distant relative is with *A. arborescens* (MF462298.1), which showed the greatest genetic distance 0.0032 (Table 3).

**Table 3:** Matrix of pairwise genetic distances among *Alternaria* taxa based on internal transcribed spacer (*ITS*) gene sequences, in addition to the out group

	**1**	**2**	**3**	**4**	**5**	**6**	**7**	**8**	**9**	**10**	**11**	**12**	**13**
PV050363.1:_Alternaria_tenuissima_isolate_ADW-5_(AUMC_16518)		0.0000	0.0000	0.0018	0.0019	0.0019	0.0019	0.0027	0.0032	0.0027	0.0248	0.0357	0.0282
PP411761.1:_Alternaria_tenuissima_isolate_Shine-Muscat_b3	0.0000		0.0000	0.0018	0.0019	0.0019	0.0019	0.0027	0.0032	0.0027	0.0248	0.0357	0.0282
KU743903.1:_Alternaria_brassicae	0.0000	0.0000		0.0018	0.0019	0.0019	0.0019	0.0027	0.0032	0.0027	0.0248	0.0357	0.0282
OR672745.1:_Alternaria_alternata_isolate_LJ1	0.0019	0.0019	0.0019		0.0027	0.0027	0.0027	0.0019	0.0032	0.0032	0.0249	0.0357	0.0282
PV341179.1:_Alternaria_alstroemeriae_isolate_S13	0.0019	0.0019	0.0019	0.0038		0.0028	0.0028	0.0034	0.0038	0.0033	0.0246	0.0355	0.0282
PP385258.1:_Alternaria_angustiovoidea_strain_SD06D04	0.0019	0.0019	0.0019	0.0038	0.0038		0.0027	0.0033	0.0026	0.0033	0.0252	0.0355	0.0282
ON790495.1:_Alternaria_compacta_isolate_63third_week	0.0019	0.0019	0.0019	0.0038	0.0038	0.0038		0.0018	0.0038	0.0034	0.0251	0.0357	0.0282
OK285209.1:_Alternaria_burnsii_isolate_BJ8	0.0038	0.0038	0.0038	0.0019	0.0057	0.0057	0.0019		0.0038	0.0038	0.0252	0.0357	0.0282
MF462298.1:_Alternaria_arborescens_strain_A.arb4	0.0057	0.0057	0.0057	0.0057	0.0076	0.0038	0.0076	0.0076		0.0038	0.0253	0.0355	0.0282
JF491200.1:_Alternaria_solani_isolate_OTA57	0.0038	0.0038	0.0038	0.0057	0.0057	0.0057	0.0057	0.0076	0.0076		0.0249	0.0356	0.0282
MT937069.1:_Fusarium_poae_strain_LCH056	0.1852	0.1852	0.1852	0.1855	0.1824	0.1881	0.1881	0.1884	0.1910	0.1879		0.0368	0.0232
MN644603.1:_Fusarium_equiseti_voucher_research_collection_Farrer_lab_126	0.2976	0.2976	0.2976	0.2976	0.2940	0.2940	0.2976	0.2976	0.2940	0.2973	0.2985		0.0342
MG438283.1:_Fusarium_fujikuroi_isolate_EU056_5.8S	0.2107	0.2107	0.2107	0.2107	0.2107	0.2107	0.2107	0.2107	0.2107	0.2107	0.1739	0.2625	

#### *Trichoderma hamatum* AUMC 16511

##### Sequence

A 570bp fragment (GenBank accession PV050359.1) was identified with a high G+C content (55.09%).

##### Phylogenetic analysis

It confirmed that *T. hamatum* established a sister clade with *T. hamatum* (KC403936.1) from 4species of the *Trichoderma* family were identified, with three species of the Mucorales order acting as an out-group (Table 4 and Fig. 2c, d).

**Table 4:** Taxa and GenBank accession numbers for the phylogenetic analysis of *Trichoderma hamatum* based on ITS sequences

No	Species	Accession number
1	*Trichoderma hamatum*	PV050359.1
2	*Trichoderma hamatum*	KC403936.1
3	*Trichoderma yunnanense*	PV202392.1
4	*Trichoderma viride*	PQ510472.1
5	*Trichoderma asperellum*	PQ787574.1
6	*Rhizopus arrhizus*	PP921273.1
7	*Rhizopus stolonifer*	MF596023.1
8	*Actinomucor elegans*	MT449966.1

##### Genetic distance

It is grouped closely with *T. hamatum* (KC403936.1) with a genetic distance of 0.0026. While the most distant relative with *T. yunnanense* (PV202392.1) had the largest genetic distance (0.0036) data are shown in Table 5.

**Table 5:** Matrix of pairwise genetic distances among *Trichoderma* taxa based on internal transcribed spacer (ITS) gene sequences, in addition to the out group

	1	2	3	4	5	6	7	8
PV050359.1:_Trichoderma_hamatum_isolate_ADW-1_(AUMC_16511)		0.0026	0.0036	0.0031	0.0031	0.0384	0.0301	0.0247
KC403936.1:_Trichoderma_hamatum_strain_THM1	0.0035		0.0037	0.0037	0.0031	0.0386	0.0301	0.0251
PV202392.1:_Trichoderma_yunnanense_isolate_SPT-2	0.0071	0.0071		0.0040	0.0031	0.0395	0.0305	0.0247
PQ510472.1:Trichoderma_viride_isolate_T6	0.0053	0.0071	0.0089		0.0036	0.0383	0.0296	0.0251
PQ787574.1:_Trichoderma_asperellum_strain_Ft602	0.0053	0.0053	0.0053	0.0071		0.0381	0.0307	0.0247
PP921273.1:_Rhizopus_arrhizus_isolate_SQQ3	0.3032	0.3065	0.3145	0.3048	0.2997		0.0391	0.0378
MF596023.1:_Rhizopus_stolonifer_isolate_wgm3	0.2389	0.2389	0.2451	0.2336	0.2457	0.3184		0.0282
MT449966.1:_Actinomucor_elegans_strain_ZZZJ30	0.1739	0.1769	0.1739	0.1773	0.1739	0.2783	0.2067	

#### *Fusarium oxysporum* AUMC 16512

##### Sequence

A 511bp fragment (GenBank accession PV050366.1) was sequenced, showing a nearly equal balance between A+T (50.88%) and G+C content.

##### Phylogenetic analysis

It was indicated that a direct relationship with *F. oxysporum* established a sister clade with *F. oxysporum* (KY008461.1) from 6species of the *Fusarium* family were identified, with three species of the Pleosporales order acting as an out-group (Table 6 and Fig. 2e, f).

**Table 6:** Taxa and GenBank accession numbers for the phylogenetic analysis of *Fusarium oxysporum* based on ITS sequences

No	Species	Accession number
1	*Fusarium oxysporum*	PV050366.1
2	*Fusarium oxysporum*	MT997883.1
3	*Fusarium oxysporum*	KY008461.1
4	*Fusarium equiseti*	JN596252.1
5	*Fusarium foetens*	OP163606.1
6	*Fusarium incarnatum*	MT003065.1
7	*Fusarium solani*	OP363852.1
8	*Alternaria porri*	PP067856.1
9	*Phoma herbarum*	JX867228.1
10	*Bipolaris sorokiniana*	MH209065.1

##### Genetic distance

It was most closely related to *F. oxysporum* (KY008461.1), with a genetic distance of 0.0021. However, the most distant relative with *F. solani* (OP363852.1) was the most genetically distant, 0.0055 (Table 7).

**Table 7:** Matrix of pairwise genetic distances among *Fusarium oxysporum* taxa and 7 related species based on internal transcribed spacer (ITS) gene sequences, in addition to the out group

	1	2	3	4	5	6	7	8	9	10
PV050366.1_Fusarium_oxysporum_isolate_ADW-8_(AUMC_16512)		0.0040	0.0042	0.0020	0.0039	0.0044	0.0055	0.0428	0.0319	0.0262
MT997883.1:_Fusarium_oxysporum_strain_ZHH-WZW-4	0.0080		0.0039	0.0020	0.0028	0.0035	0.0048	0.0418	0.0306	0.0269
KY008461.1:_Fusarium_oxysporum_strain_FLN-2	0.0079	0.0080		0.0029	0.0034	0.0040	0.0052	0.0422	0.0314	0.0274
JN596252.1:_Fusarium_equiseti_culture-collection_MTCC:1978	0.0020	0.0020	0.0041		0.0020	0.0000	0.0029	0.0404	0.0306	0.0261
OP163606.1:_Fusarium_foetens_isolate_SB-PH–S-15	0.0079	0.0040	0.0059	0.0020		0.0019	0.0039	0.0422	0.0311	0.0266
MT003065.1:_Fusarium_incarnatum_isolate_SRRB-54	0.0102	0.0061	0.0081	0.0000	0.0020		0.0000	0.0443	0.0323	0.0268
OP363852.1:_Fusarium_solani_strain_F5	0.0160	0.0120	0.0140	0.0041	0.0079	0.0000		0.0435	0.0321	0.0271
PP067856.1:_Alternaria_porri_isolate_AP1	0.3512	0.3434	0.3477	0.3335	0.3466	0.3616	0.3597		0.0416	0.0515
JX867228.1:_Phoma_herbarum_strain_JL-23	0.2498	0.2366	0.2459	0.2323	0.2424	0.2526	0.2531	0.3475		0.0425
MH209065.1:_Bipolaris_sorokiniana_strain_D6899	0.1947	0.2029	0.2050	0.1868	0.1977	0.1937	0.2042	0.4115	0.3360	

#### *Fusarium solani* AUMC 16514

##### Sequence

A 536bp fragment (GenBank accession PV050365.1) was obtained, which had a high G+C content (53.92%).

##### Phylogenetic analysis

The analysis confirmed a close relationship with *F. solani* (OL314655.1) from 5species of the *Fusarium* family were identified, with three species of the Pleosporales order acting as an out-group (Table 8 and Fig. 2g, h).

**Table 8:** Taxa and GenBank accession numbers for the phylogenetic analysis of *Fusarium solani* based on ITS sequences

No	Species	Accession number
1	*Fusarium solani*	PV050365.1
2	*Fusarium solani*	OL314655.1
3	*Fusarium haematococcum*	KJ780750.1
4	*Fusarium keratoplasticum*	LC745689.1
5	*Fusarium equiseti*	MN452325.1
6	*Fusarium incarnatum*	DQ307676.1
7	*Palmiascoma qujingense*	PQ032321.1
8	*Didymella* sp.	OQ130554.1
9	*Phaeosphaeria* sp.	OR810383.1

##### Genetic distance

The analysis confirmed a close relationship with *F. solani* (OL314655.1), showing a genetic distance of 0.0033, but the greatest genetic distance (0.0115) was to *F. incarnatum* (DQ307676.1), data show in Table 9.

**Table 9:** Matrix of pairwise genetic distances among *Fusarium solani* taxa and 5 related species based on internal transcribed spacer (ITS) gene sequences, in addition to the out group

	1	2	3	4	5	6	7	8	9
PV050365.1:_Fusarium_solani_isolate_ADW-7_(AUMC_16514)		0.0033	0.0062	0.0090	0.0062	0.0115	0.0331	0.0336	0.0343
OL314655.1:_Fusarium_solani_strain_GXRST33	0.0058		0.0049	0.0071	0.0062	0.0112	0.0321	0.0330	0.0346
KJ780750.1:_Fusarium_haematococcum_isolate_A1S2-D11	0.0191	0.0116		0.0056	0.0058	0.0095	0.0301	0.0317	0.0345
LC745689.1:_Fusarium_keratoplasticum_sample171	0.0356	0.0236	0.0155		0.0063	0.0096	0.0300	0.0316	0.0337
MN452325.1:_Fusarium_equiseti_isolate_W_IASO2_2_1	0.0171	0.0171	0.0150	0.0194		0.0101	0.0339	0.0301	0.0333
DQ307676.1:_Fusarium_incarnatum	0.0501	0.0478	0.0366	0.0387	0.0403		0.0352	0.0338	0.0365
PQ032321.1:_Palmiascoma_qujingense_isolate_B65-1	0.2584	0.2511	0.2341	0.2345	0.2645	0.2716		0.0361	0.0451
OQ130554.1:_Didymella_sp._breed_9-3	0.2555	0.2517	0.2379	0.2389	0.2231	0.2477	0.2756		0.0397
OR810383.1:_Phaeosphaeria_sp._isolate_491	0.2800	0.2840	0.2806	0.2728	0.2583	0.2846	0.3670	0.3149	

#### *Fusarium equiseti* AUMC 16515

##### Sequence

A 509bp fragment (GenBank accession PV050360.1) was identified with a balanced A+T (50.10%) and G+C content.

##### Phylogenetic analysis

The analysis indicated that the *F. equiseti* (OR654240.1) was sister to a clade with our sample from 15 species of the *Fusarium* family were identified, with three species of the Pleosporales order acting as an out-group (Table 10 and Fig. 2i, j).

**Table 10:** Taxa and GenBank accession numbers for the phylogenetic analysis of *Fusarium equiseti* based on ITS sequences

No	Species	Accession number
1	*Fusarium equiseti*	PV050360.1
2	*Fusarium equiseti isolate*	OR654240.1
3	*Fusarium chlamydosporum*	MN480498.1
4	*Fusarium coffeatum*	MT742819.1
5	*Fusarium camptoceras*	AB820722.1
6	*Fusarium tanahbumbuense*	MW016553.1
7	*Fusarium humuli*	MW016529.1
8	*Fusarium guilinense*	PV954806.1
9	*Fusarium verticillioides*	OR485023.1
10	*Fusarium hainanense*	MW016528.1
11	*Fusarium incarnatum*	MN871562.1
12	*Fusarium irregulare*	MW365369.1
13	*Fusarium clavum*	PQ782217.1
14	*Fusarium sp.*	OQ421989.1
15	*Fusarium croceum*	PP947369.1
16	*Fusarium oxysporum*	MW063667.1
17	*Paraconiothyrium cyclothyrioides*	MN644708.1
18	*Bipolaris sorokiniana*	MH209065.1
19	*Hendersonia sacchari*	MH855416.1

##### Genetic distance

It was most closely related to *F. equiseti* (OR654240.1) and *F. humuli* (MW016529.1), both with a genetic distance of 0.0019. While the most distant relative with *F. irregulare* (MW365369.1) was the most distant, 0.0058 (Table 11).

**Table 11:** Matrix of pairwise genetic distances among *Fusarium equiseti* taxa and 15 related species based on internal transcribed spacer (ITS) gene sequences, in addition to the out group

	1	2	3	4	5	6	7	8	9	10	11	12	13	14	15	16	17	18	19
PV050360.1:_Fusarium_equiseti_isolate_ADW-2_(AUMC_16515)		0.0019	0.0028	0.0041	0.0041	0.0034	0.0019	0.0056	0.0034	0.0028	0.0034	0.0058	0.0028	0.0045	0.0045	0.0045	0.0230	0.0287	0.0300
OR654240.1:_Fusarium_equiseti_isolate_DJMZJ009	0.0020		0.0020	0.0045	0.0045	0.0028	0.0000	0.0052	0.0028	0.0020	0.0028	0.0062	0.0034	0.0041	0.0048	0.0049	0.0230	0.0283	0.0302
MN480498.1:_Fusarium_chlamydosporum_isolate_MAOS2	0.0040	0.0020		0.0041	0.0041	0.0019	0.0000	0.0049	0.0028	0.0020	0.0028	0.0058	0.0034	0.0036	0.0044	0.0044	0.0227	0.0279	0.0296
MT742819.1:_Fusarium_coffeatum_isolate	0.0079	0.0100	0.0080		0.0000	0.0044	0.0041	0.0044	0.0040	0.0045	0.0040	0.0045	0.0028	0.0019	0.0020	0.0019	0.0216	0.0278	0.0286
AB820722.1:_Fusarium_camptoceras_strain:_CBS_193.65	0.0079	0.0100	0.0080	0.0000		0.0044	0.0041	0.0044	0.0040	0.0045	0.0040	0.0045	0.0028	0.0019	0.0020	0.0019	0.0216	0.0278	0.0286
MW016553.1:_Fusarium_tanahbumbuense_strain_LC13726	0.0059	0.0040	0.0020	0.0100	0.0100		0.0019	0.0052	0.0034	0.0028	0.0034	0.0055	0.0039	0.0040	0.0048	0.0041	0.0229	0.0281	0.0293
MW016529.1:_Fusarium_humuli_strain_LC13702	0.0020	0.0000	0.0000	0.0080	0.0080	0.0020		0.0049	0.0021	0.0000	0.0021	0.0058	0.0034	0.0036	0.0044	0.0045	0.0228	0.0280	0.0297
PV954806.1:_Fusarium_guilinense_isolate_APFu-25	0.0162	0.0141	0.0121	0.0100	0.0100	0.0141	0.0121		0.0048	0.0053	0.0048	0.0057	0.0034	0.0039	0.0048	0.0048	0.0232	0.0289	0.0279
OR485023.1:_Fusarium_verticillioides_isolate_f3	0.0059	0.0040	0.0040	0.0080	0.0080	0.0059	0.0020	0.0121		0.0021	0.0000	0.0062	0.0034	0.0034	0.0044	0.0044	0.0229	0.0281	0.0305
MW016528.1:_Fusarium_hainanense_strain_LC13701	0.0040	0.0020	0.0020	0.0100	0.0100	0.0040	0.0000	0.0142	0.0020		0.0021	0.0062	0.0034	0.0041	0.0049	0.0049	0.0231	0.0281	0.0303
MN871562.1:_Fusarium_incarnatum_clone_LS139	0.0059	0.0040	0.0040	0.0080	0.0080	0.0059	0.0020	0.0121	0.0000	0.0020		0.0062	0.0034	0.0034	0.0044	0.0044	0.0229	0.0281	0.0305
MW365369.1:_Fusarium_irregulare_strain_VTCC:31,704	0.0161	0.0182	0.0161	0.0100	0.0100	0.0141	0.0161	0.0162	0.0182	0.0182	0.0182		0.0034	0.0049	0.0048	0.0041	0.0226	0.0289	0.0280
PQ782217.1:_Fusarium_clavum_strain_RR22(I)/2.3	0.0040	0.0060	0.0060	0.0040	0.0040	0.0081	0.0060	0.0061	0.0060	0.0061	0.0060	0.0060		0.0034	0.0020	0.0034	0.0225	0.0282	0.0271
OQ421989.1:_Fusarium_sp._breed_MHUB11408	0.0100	0.0079	0.0059	0.0020	0.0020	0.0079	0.0060	0.0080	0.0059	0.0080	0.0059	0.0120	0.0060		0.0027	0.0027	0.0216	0.0274	0.0288
PP947369.1:_Fusarium_croceum_strain_CN104E8	0.0100	0.0120	0.0100	0.0020	0.0020	0.0120	0.0100	0.0121	0.0100	0.0120	0.0100	0.0120	0.0020	0.0040		0.0027	0.0216	0.0281	0.0283
MW063667.1:_Fusarium_oxysporum_f._sp._ricini_isolate_For-12–2	0.0100	0.0120	0.0100	0.0020	0.0020	0.0080	0.0100	0.0121	0.0100	0.0120	0.0100	0.0080	0.0060	0.0040	0.0040		0.0218	0.0280	0.0283
MN644708.1:_Paraconiothyrium_cyclothyrioides_voucher_research_collection_Farrer_lab_66	0.1596	0.1596	0.1566	0.1475	0.1475	0.1596	0.1570	0.1612	0.1566	0.1602	0.1566	0.1629	0.1535	0.1475	0.1475	0.1504		0.0283	0.0313
MH209065.1:_Bipolaris_sorokiniana_strain_D6899	0.2295	0.2260	0.2227	0.2225	0.2225	0.2260	0.2233	0.2284	0.2227	0.2233	0.2227	0.2301	0.2205	0.2191	0.2259	0.2259	0.2116		0.0434
MH855416.1:_Hendersonia_sacchari_culture_CBS:203.33	0.2386	0.2423	0.2389	0.2318	0.2318	0.2355	0.2395	0.2303	0.2464	0.2426	0.2464	0.2285	0.2151	0.2355	0.2284	0.2284	0.2343	0.3675	

#### *Fusarium incarnatum* AUMC 16516

##### Sequence

A 504bp fragment (GenBank accession PV050361.1) was sequenced, having a balanced A+T and G+C content (50.00% each).

##### Phylogenetic analysis

It showed a direct relationship with *F. incarnatum* (MH345928.1), which was sister to a clade with our sample from 8species of the *Fusarium* family were identified, with three species of the Pleosporales order acting as an out-group (Table 12 and Fig. 2k, l)

**Table 12:** Taxa and GenBank accession numbers for the phylogenetic analysis of *Fusarium incarnatum* based on ITS sequences

No	Species	Accession number
1	*Fusarium incarnatum*	PV050361.1
2	*Fusarium incarnatum*	MH345928.1
3	*Fusarium solani*	PQ855922.1
4	*Fusarium clavum*	PQ782217.1
5	*Fusarium humuli*	MW016529.1
6	*Fusarium equiseti*	KU752196.1
7	*Fusarium chlamydosporum*	MK212882.1
8	*Tricholoma matsutake*	MT448907.1
9	*Fusarium sp.*	OL627343.1
10	*Purpureocillium lilacinum*	OQ732728.1
11	*Uncultured Ophiocordyceps*	MK407252.1
12	*Ophiocordyceps sinensis*	KX218227.1

##### Genetic distance

It showed the smallest genetic distance (0.0020) to *F. chlamydosporum* (MK212882.1), but *F. solani* (PQ855922.1) was the most distant relative in the comparison, with 0.0046 (Table 13).

**Table 13:** Matrix of pairwise genetic distances among *Fusarium incarnatum* taxa and 8 related species based on internal transcribed spacer (ITS) gene sequences, in addition to the out group

	1	2	3	4	5	6	7	8	9	10	11	12
PV050361.1:_Fusarium_incarnatum_isolate_ADW-3_(AUMC_16516)		0.0021	0.0046	0.0030	0.0027	0.0028	0.0020	0.0027	0.0027	0.0100	0.0152	0.0157
MH345928.1:_Fusarium_incarnatum_isolate_88	0.0020		0.0046	0.0030	0.0035	0.0036	0.0030	0.0035	0.0035	0.0096	0.0155	0.0161
PQ855922.1:_Fusarium_solani_strain_XJF6-12	0.0101	0.0101		0.0035	0.0044	0.0035	0.0035	0.0050	0.0050	0.0115	0.0167	0.0173
PQ782217.1:_Fusarium_clavum_strain_RR22(I)/2.3	0.0040	0.0040	0.0060		0.0028	0.0000	0.0000	0.0035	0.0035	0.0106	0.0157	0.0163
MW016529.1:_Fusarium_humuli_strain_LC13702	0.0040	0.0060	0.0101	0.0040		0.0028	0.0028	0.0000	0.0000	0.0100	0.0149	0.0155
KU752196.1:_Fusarium_equiseti_isolate_F6	0.0040	0.0060	0.0061	0.0000	0.0040		0.0000	0.0028	0.0028	0.0104	0.0155	0.0161
MK212882.1:_Fusarium_chlamydosporum_isolate_P048	0.0020	0.0040	0.0061	0.0000	0.0040	0.0000		0.0028	0.0028	0.0104	0.0155	0.0161
MT448907.1:_Tricholoma_matsutake_isolate_TWD25	0.0040	0.0060	0.0122	0.0060	0.0000	0.0040	0.0040		0.0000	0.0100	0.0149	0.0154
OL627343.1:_Fusarium_sp._strain_A	0.0040	0.0060	0.0122	0.0060	0.0000	0.0040	0.0040	0.0000		0.0100	0.0149	0.0154
OQ732728.1:_Purpureocillium_lilacinum_strain_ZTd-R-ENDO-18A	0.0424	0.0403	0.0538	0.0469	0.0425	0.0448	0.0448	0.0424	0.0424		0.0160	0.0160
MK407252.1:_Uncultured_Ophiocordyceps_clone_D1479ITS	0.0948	0.0975	0.1079	0.0998	0.0924	0.0977	0.0977	0.0922	0.0922	0.0979		0.0036
KX218227.1:_Ophiocordyceps_sinensis_clone_2015GM1-14–2013	0.1002	0.1029	0.1133	0.1053	0.0977	0.1032	0.1032	0.0975	0.0975	0.0978	0.0061	

#### *Purpureocillium lilacinum* AUMC 16504

##### Sequence

A 555bp fragment (GenBank accession PV050362.1) was identified with balanced A+T and G+C content (50.00% each).

##### Phylogenetic analysis

It confirmed that *P. lilacinum* established a sister clade with *P. lilacinum* (PP385054.1) from 4species of the *Purpureocillium* family were identified, with three species of the Pleosporales order acting as an out-group (Table 14 and Fig. 2m, n).

**Table 14:** Taxa and GenBank accession numbers for the phylogenetic analysis of *Purpureocillium lilacinum* based on *ITS *sequences

No	Species	Accession number
1	*Purpureocillium lilacinum*	PV050362.1
2	*Purpureocillium lilacinum*	PP385054.1
3	*Purpureocillium takamizusanense*	MT568631.1
4	*Purpureocillium lavendulum*	MW113502.1
5	*Purpureocillium sodanum*	PP384647.1
6	*Fusarium incarnatum*	MW450597.1
7	*Yarrowia lipolytica*	OR398955.1
8	*Trichomonascus ciferrii*	MT410704.1

##### Genetic distance

It is grouped closely with *P. lilacinum* (PP385054.1) at a genetic distance of 0.0026. While the largest genetic distance (0.0240) was observed with *P. sodanum* (PP384647.1), according to Table 15.

**Table 15:** Matrix of pairwise genetic distances among *Purpureocillium* taxa based on internal transcribed spacer (ITS) gene sequences, in addition to the out group

	1	2	3	4	5	6	7	8
PV050362.1:_Purpureocillium_lilacinum_isolate_ADW-4_(AUMC_16504)		0.0026	0.0056	0.0084	0.0240	0.0260	0.0363	0.0552
PP385054.1:_Purpureocillium_lilacinum_strain_JX11A04	0.0036		0.0047	0.0082	0.0233	0.0255	0.0357	0.0548
MT568631.1:_Purpureocillium_takamizusanense_isolate_RCEF5016	0.0151	0.0112		0.0085	0.0228	0.0261	0.0361	0.0562
MW113502.1:_Purpureocillium_lavendulum_strain_SC45B05	0.0345	0.0325	0.0333		0.0276	0.0261	0.0373	0.0532
PP384647.1:Purpureocillium_sodanum_strain_AH07D04	0.1820	0.1765	0.1705	0.2180		0.0443	0.0498	0.0922
MW450597.1:_Fusarium_incarnatum_strain_FE11	0.1885	0.1823	0.1892	0.1924	0.3727		0.0375	0.0590
OR398955.1:_Yarrowia_lipolytica_strain_YZK8	0.3000	0.2921	0.2930	0.3055	0.4281	0.2900		0.0641
MT410704.1:_Trichomonascus_ciferrii_isolate_ZC-9	0.4773	0.4773	0.4836	0.4618	0.7888	0.4941	0.5459	

#### *Geotrichum candidum* AUMC 16517

##### Sequence

A 332bp fragment (GenBank accession PV050364.1) was sequenced, showing a very high A+T content (65.36%).

##### Phylogenetic analysis

It was indicated that a direct relationship with *G. candidum* established a sister clade with *G. candidum* (MT071788.1) 5species of the *Geotrichum* family were identified, with three species of the Hypocreales order acting as an out-group (Table 16 and Fig. 2o, p).

**Table 16:** Taxa and GenBank accession numbers for the phylogenetic analysis of *Geotrichum candidum* based on *ITS* sequences

No	Species	Accession number
1	*Geotrichum candidum*	PV050364.1
2	*Geotrichum candidum*	MT071788.1
3	*Geotrichum silvicola*	PQ044650.1
4	*Geotrichum bryndzae*	KP132252.1
5	*Galactomyces geotrichum*	HG532089.1
6	*Geotrichum galactomycetum*	KY315925.1
7	*Beauveria bassiana*	OP028906.1
8	*Fusarium virguliforme*	MZ854209.1
9	*Beauveria caledonica*	KY471655.1

##### Genetic distance

The analysis revealed it was genetically identical (distance of 0.0000) to *G. candidum* (MT071788.1). However, the *G. silvicola* (PQ044650.1) was the most distant, with a genetic distance of 0.0079 (Table 17).

**Table 17:** Matrix of pairwise genetic distances among *Geotrichum* taxa based on internal transcribed spacer (*ITS*) gene sequences, in addition to the out group

	1	2	3	4	5	6	7	8	9
PV050364.1:_Geotrichum_candidum_isolate_ADW-6_(AUMC_16517)		0.0000	0.0079	0.0070	0.0070	0.0070	0.1776	0.2063	0.1802
MT071788.1:_Geotrichum_candidum_strain_DY1-2	0.0000		0.0079	0.0070	0.0070	0.0070	0.1776	0.2063	0.1802
PQ044650.1:_Geotrichum_silvicola_isolate_14c-4	0.0188	0.0188		0.0045	0.0045	0.0045	0.1692	0.1868	0.1770
KP132252.1:_Geotrichum_bryndzae_strain_PMM10-1197L	0.0154	0.0154	0.0062		0.0000	0.0000	0.1712	0.1928	0.1752
HG532089.1:_Galactomyces_geotrichum_strain_ABM5136	0.0154	0.0154	0.0062	0.0000		0.0000	0.1712	0.1928	0.1752
KY315925.1:_Geotrichum_galactomycetum_voucher_PLP847_a	0.0154	0.0154	0.0062	0.0000	0.0000		0.1712	0.1928	0.1752
OP028906.1:_Beauveria_bassiana_isolate_CTUHT-6	1.0252	1.0252	0.9974	1.0025	1.0025	1.0025		0.0444	0.0198
MZ854209.1:_Fusarium_virguliforme_isolate_HQ-8	1.1244	1.1244	1.0412	1.0557	1.0557	1.0557	0.2701		0.0407
KY471655.1:_Beauveria_caledonica_isolate_Bea110	1.0452	1.0452	1.0151	1.0134	1.0134	1.0134	0.1013	0.2439	

### Detection of enzyme activity for the different isolates

**Fig. 3:**
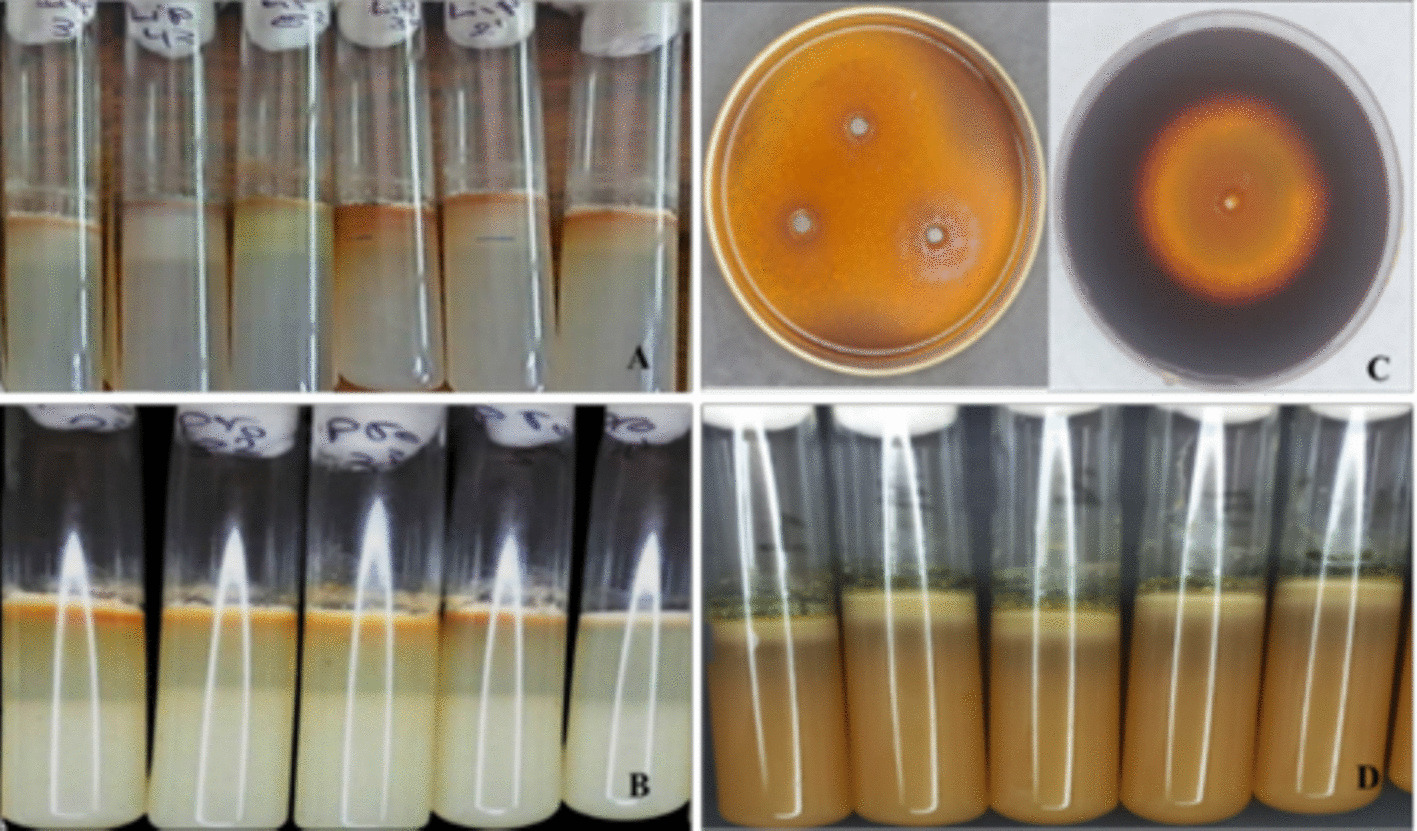
Detection of enzyme activity for local fungal isolates: **A**, Lipase activity; **B**, Protease activity; **C**, Chitinase activity, and **D**, Phospholipase activity

**Fig. 4:**
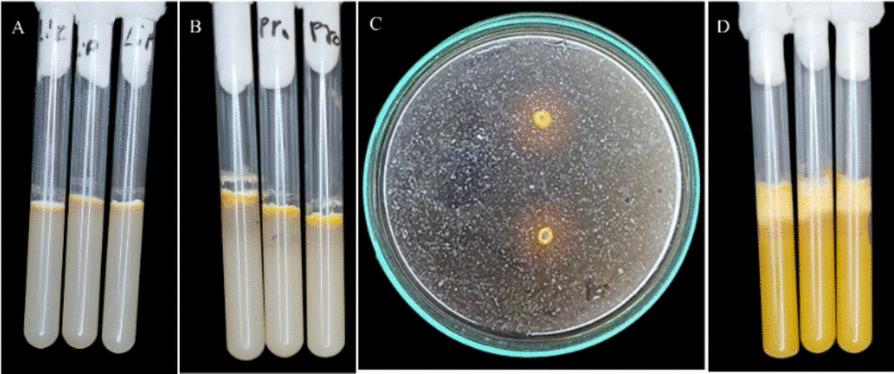
Detection of enzyme activity for positive control (*Beauveria bassiana*): **A**, Lipase activity; **B**, Protease activity; **C**, Chitinase activity, and **D**, Phospholipase activity

**Fig. 5:**
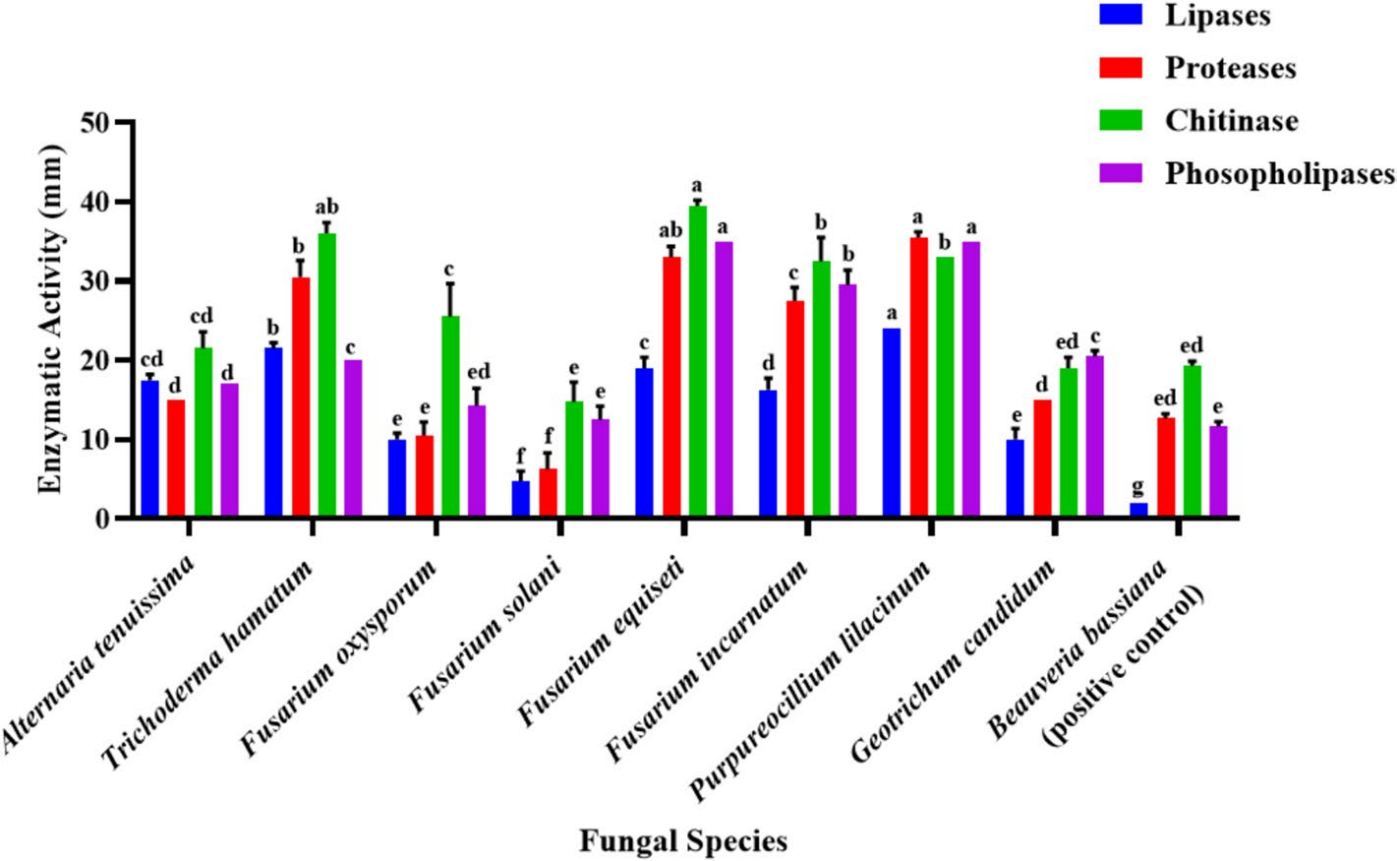
The average of extracellular enzyme activity for 22 fungal isolates belongs to eight fungal species, and the positive control (*Beauveria bassiana*)

Data in Table 18, Figs. 3, 4, and 5 indicate that all 22 fungal isolates belonging to 8 species shared the ability to secrete the four essential enzymes compared with *Beauveria bassiana* AUMC17426 as a positive control. Statistical analysis of secretion magnitudes indicated that the significant differences were further confirmed by the high F-values recorded for all enzymes: Lipases (F = 132.50^**^), Proteases (F = 136.06^**^), Chitinase (F = 29.70^**^), and Phospholipases (F = 102.24^**^). Regarding the specific enzymatic peaks, *Fusarium equiseti* (AUMC 16501) achieved the absolute maximum secretion of chitinase (39.50±0.71mm), significantly outperforming all other isolates. While *Purpureocillium lilacinum* (AUMC 16504) was uniquely positioned in the top statistical rank for most enzymes, its peak performance was most notable in lipase (24.00±0.00mm), protease (35.50±0.71mm), and phospholipases(35.00±0.00mm). Furthermore, *Trichoderma hamatum* exhibited a competitive edge through its robust triple secretion profile, marking high activities in protease (30.50±2.12mm), chitinase (36.00±1.41mm), and phospholipase (20.00±0.00mm). This was followed by *Fusarium incarnatum*, which emerged as a robust producer of phospholipase (29.50±1.91mm). Occupying a middle ground, isolates such as *F. oxysporum*, *Alternaria tenuissima*, and *G. candidum* displayed fluctuating activities, with chitinase secretion with average of 25.50±4.20mm, 21.50±2.12mm, and 19.00±1.41mm, respectively. In stark contrast, the lowest enzymatic activity across all tested categories was recorded for *Fusarium solani*, which yielded the study's minimum values, including a protease zone of only 6.25±2.06mm. Concerning the enzymatic activity of the positive control (*Beauveria bassiana*) yielded 12.67±0.58mm, 19.33±0.58mm, and 11.67±0.58mm for Lipases, Proteases, Chitinase, and Phospholipases, 2.00±0.00mm, respectively (Fig. 4).

**Table 18:** Detection of enzyme activity: Lipases, Proteases, Chitinase, and Phospholipases for 22 fungal isolates belonging to eight fungal species and a positive control (*Beauveria bassiana*)

Fungal isolates		Detection of enzymes activity			
		**Lipases**	**Proteases**	**Chitinase**	**Phospholipases**
***Alternaria tenuissima***	**1st**	18	17	20	15
	**2nd**	17	17	23	15
Mean ± SD		17.50 ± 0.71^ cd^	15.00 ± 0.00^d^	21.50±2.12^ cd^	17.00 ± 0.00^d^
***Trichoderma hamatum***	**1st**	22	20	37	32
	**2nd**	21	20	35	29
Mean ± SD		21.50 ± 0.71^b^	30.50 ± 2.12^b^	36.00 ± 1.41^ab^	20.00 ± 0.00^c^
***Fusarium oxysporum***	**1st**	11	15	20	9
	**2nd**	10	11	30	12
	**3rd**	10	16	25	12
	**4th**	9	15	27	9
Mean ± SD		10.00 ± 0.82^e^	10.50 ± 1.73^e^	25.50 ± 4.20^c^	14.25 ± 2.22^ed^
***Fusarium solani***	**1st**	6	14	18	8
	**2nd**	5	14	15	8
	**3rd**	5	11	14	5
	**4th**	3	11	12	4
Mean ± SD		4.75 ± 1.26^f^	6.25 ± 2.06^f^	14.75 ± 2.50^e^	12.50 ± 1.73^e^
***Fusarium equiseti***	**1st**	20	35	40	34
	**2nd**	18	35	39	32
Mean ± SD		19.00 ± 1.41^c^	33.00 ± 1.41^ab^	39.50 ± 0.71^a^	35.00 ± 0.00^a^
***Fusarium incarnatum***	**1st**	17	32	36	29
	**2nd**	18	28	30	28
	**3rd**	15	28	30	25
	**4th**	15	30	34	28
Mean ± SD		16.25 ± 1.50^d^	27.50 ± 1.73^c^	32.50 ± 3.00^b^	29.50 ± 1.91^b^
***Purpureocillium lilacinum***	**1st**	24	35	33	35
	**2nd**	24	35	33	36
Mean ± SD		24.00 ± 0.00^a^	35.50 ± 0.71^a^	33.00 ± 0.00^b^	35.00 ± 0.00^a^
***Geotrichum candidum***	**1st**	11	15	20	21
	**2nd**	9	15	18	20
Mean ± SD		10.00 ± 1.41^e^	15.00 ± 0.00^d^	19.00 ± 1.41^ed^	20.50 ± 0.71^c^
***Beauveria bassiana* (positive control)**	**R1**^**@**^	2	13	19	12
	**R2**	2	13	19	12
	**R3**	2	12	20	11
Mean ± SD		2.00 ± 0.00^g^	12.67 ± 0.58^ed^	19.33 ± 0.58^ed^	11.67 ± 0.58^e^
**F-value**		132.50^**^	136.06^**^	29.70^**^	102.24^**^

^*^ = significant at 0.05 level of probability, ** = highly significant at 0.01 level of probability, and ns = Insignificant

@ R = (i.e., 3 replicates)

^a-g^ Means within the same letter in each column is not significant at a 5% level of probability, according to Duncan’s multiple range test with Kramer’s adjustment

## Discussion

The current study focuses on discovering and extracting local isolates adapted to the environmental conditions of Assiut, Egypt. A total of 22 fungal isolates belonging to 8 species associated with mosquitoes were isolated from agricultural drainage water in Assiut. In general, locally isolated species are often more tolerant of local environmental conditions, making them more effective in integrated pest management (IPM), as emphasized by Shahriari et al. [24].

Regarding the molecular identification of the above-mentioned species studied, it indicated a full genetic identity that matches the same species in GenBank. However, *Alternaria tenuissima* showed close genetic proximity to other *Alternaria* species, such as *A. brassica*. The distinct morphological beak measurements recorded in our results indicated clear discrimination between both species according to Simmons [25]. Both identification approaches address the limitations of using single molecular markers for closely related taxa and provide a reliable taxonomic baseline for future field applications.

Concerning extracellular enzymes (proteases, lipases, chitinases, and phospholipases) are generally recognized as fundamental biochemical "tools" for penetrating the insect cuticle, as reported by Sari et al. [23]. The entomopathogenic fungi, particularly *Beauveria bassiana*, are one of the most studied and productive species worldwide and are among the greatest biological agents for pest control. The enzymatic arsenal that the fungus secretes to break through the insect's chitinous hardness is what gives it its power. A combination of lipases, chitinases, and proteases is released [11].

The initial results from screening the studied isolates indicate a diversity in enzyme secretion. The continuous secretion of these enzymes suggests their potential to induce insect pathogens.

Therefore, in this study, *B. bassiana* was used as a positive control compared to the enzymatic potential of our local fungal isolates. The result indicated that although *B. bassiana* is the traditional and widely recognized model for biological control, the comparison reveals that other fungi in this group have raw enzymatic activity that significantly exceeds it, such as *Purpureocillium lilacinum* and *Fusarium equiseti*. This could make them excellent candidates for additional research as highly promising biological control agents, particularly in settings that call for quick penetration of biological tissues.

Also, the noteworthy enzymatic activity of *P. lilacinum* and *Fusarium equiseti* is of particular interest. This aligns with the findings of Ni et al. [18] reported that *P. lilacinum* and specific *Fusarium* species, particularly *F. verticillioides,* possess potent larvicidal and egg-killing properties against Diptera pests. By identifying similar enzymatic properties in our local isolates, this study provides strong preliminary evidence for their potential use as sustainable antimicrobial agents.

Moreover, the synergistic effect of the proteases and chitinases identified in *Trichoderma hamatum* and *F. incarnatum* enhances this potential, as the sequential degradation of proteases and chitin is a crucial step in the fungal infection process [23].

On the other hand, *Geotrichum candidum* is not usually categorized as a direct insect pathogen, but its isolation from mosquito nurseries in Asyut is noteworthy. Our results showed an intermediate enzymatic pattern for this isolate, AUMC 16517 (Table 18). Also, this finding is consistent with the findings of Pottier et al. [20] reported that although *G. candidum* possesses a basic protease and extracellular lipase mechanism, the expression of enzymatic levels show considerable strain-specific variability. This species probably acts as a secondary colony alongside more virulent species within the mosquito environment.

Finally, the data presented here constitute a critical preliminary selection phase. This strategic approach allows for the selection of isolates and a focused transition to future research, where the most enzymatically active candidates, such as *P. lilacinum* (AUMC 16504) and *F. equiseti* (AUMC 16515), can be subjected to rigorous bioassays to determine their pathogenic efficacy in the sustainable microbial control of *Culex pipiens* mosquitoes and other insects.

## Conclusion

The current study successfully characterized 22 local fungal isolates belonging to eight species, extracted from mosquito habitats in Asyut Governorate, Egypt. By combining detailed morphological characterization with internal DNA (ITS) sequencing, a robust taxonomic basis was established for these local species compared to global species. The diverse secretion of key lytic enzymes compared to standard species such as *Beauveria bassiana* indicates the classification of these local strains as promising agents for sustainable microbial control. So, future studies are needed to determine the peak production of these enzymes and to conduct biological assays to establish actual mortality rates and median lethal concentration (LC50) values for these identified isolates against *Culex pipiens* and other insects. Ultimately, developing sustainable biological control strategies tailored to local environmental conditions remains crucial for reducing the burden of mosquito-borne diseases while preserving the ecological integrity of the agricultural system to achieve the Sustainable Development Goals (SDGs).

## Data Availability

The datasets analyzed during the current study are available in the [GenBank/NCBI] repository, WEB LINK[https://www.ncbi.nlm.nih.gov/], under Accession Numbers: [PV050363.1](https:/www.ncbi.nlm.nih.gov/nuccore/PV050363.1), [PV050359.1](https:/www.ncbi.nlm.nih.gov/nuccore/PV050359.1), [PV050366.1](https:/www.ncbi.nlm.nih.gov/nuccore/PV050366.1), [PV050365.1](https:/www.ncbi.nlm.nih.gov/nuccore/PV050365.1), [PV050360.1](https:/www.ncbi.nlm.nih.gov/nuccore/PV050360.1), [PV050361.1](https:/www.ncbi.nlm.nih.gov/nuccore/PV050361.1), [PV050362.1](https:/www.ncbi.nlm.nih.gov/nuccore/PV050362.1), [PV050364.1](https:/www.ncbi.nlm.nih.gov/nuccore/PV050364.1)].

## Abbreviation

AUMC: Assiut University Mycological Centre
AUMMC: Assiut University Moubasher Mycological Center
A: Adenine
BLAST: Basic Local Alignment Search Tool
CTAB: Cetyltrimethylammonium Bromide
Cz: Czapek’s Dox agar
C: Cytosine
DNA: Deoxyribonucleic Acid
EDTA: Ethylenediaminetetraacetic Acid
EPF: Entomopathogenic fungi
G: Guanine
IPM: Integrated pest management
ITS1: Internal Transcribed Spacer 1
ITS4: Internal Transcribed Spacer 4
MEA: Malt extract agar
ME: Minimum Evolution
MBCAs: Microbial Biological Control Agents
OA: Oatmeal Agar
NCBI: National Centre of Biotechnology Information
NJ: Neighbor-joining
PCR: Polymerase Chain Reaction
PDA: Potato Dextose Agar
PSA: Potato Sucrose Agar
rDNA: Ribosomal DNA
SDA: Sabouraud Dextrose Agar
SDGs: Sustainable Development Goals
T: Thymine
WHO: World Health Organization
